# Induction of Apoptosis and Antitumor Activity of Eel Skin Mucus, Containing Lactose-Binding Molecules, on Human Leukemic K562 Cells

**DOI:** 10.3390/md13063936

**Published:** 2015-06-19

**Authors:** Choong-Hwan Kwak, Sook-Hyun Lee, Sung-Kyun Lee, Sun-Hyung Ha, Seok-Jong Suh, Kyung-Min Kwon, Tae-Wook Chung, Ki-Tae Ha, Young-Chae Chang, Young-Choon Lee, Dong-Soo Kim, Hyeun-Wook Chang, Cheorl-Ho Kim

**Affiliations:** 1 Department of Biological Science, Sungkyunkwan University, Chunchun-Dong 300, Jangan-Gu, Suwon City, Kyunggi-Do 440-746, Korea; E-Mails: hahaaaa@nate.com (C.-H.K.); sh880831@skku.edu (S.-H.L.); gyoon1202@hanmail.net (S.-K.L.); sunspring5@naver.com (S.-H.H.); good_stone@hanmail.net (S.-J.S.); muscaria80@naver.com (K.-M.K.); 2 Division of Applied Medicine, School of Korean Medicine, Pusan National University, Yangsan, Gyeongsangnam-Do 626-770, Korea; E-Mails: chungtw@hanmail.net (T.-W.C.); hagis@pusan.ac.kr (K.-T.H.); 3 Research Institute of Biomedical Engineering and Department of Medicine, Catholic University of Daegu School of Medicine, Daegu 705-718, Korea; E-Mail: ycchang@cu.ac.kr; 4 College of Natural Resources and Life Science, Dong-A University, Busan, 604-714, Korea; E-Mail: yclee@dau.ac.kr; 5 Department of Food Science and Biotechnology, Kyung Sung University, Nam-Gu, Busan, 608-736, Korea; E-Mail: dskim@kyungsung.ac.kr; 6 College of Pharmacy, Yeungnam University, Gyeongsan 712-749, Korea

**Keywords:** mucus, *Anguilla japonica*, apoptosis, lectin-like protein, glycan

## Abstract

For innate immune defense, lower animals such as fish and amphibian are covered with skin mucus, which acts as both a mechanical and biochemical barrier. Although several mucus sources have been isolated and studied for their biochemical and immunological functions, the precise mechanism(s) of action remains unknown. In the present study, we additionally found the eel skin mucus (ESM) to be a promising candidate for use in anti-tumor therapy. Our results showed that the viability of K562 cells was decreased in a dose-dependent manner by treatment with the isolated ESM. The cleaved forms of caspase-9, caspase-3 and poly adenosine diphosphate-ribose polymerase were increased by ESM. The levels of Bax expression and released cytochrome C were also increased after treatment with ESM. Furthermore, during the ESM mediated-apoptosis, phosphorylation levels of ERK1/2 and p38 but not JNK were increased and cell viabilities of the co-treated cells with ESM and inhibitors of ERK 1/2 or p38 were also increased. In addition, treatment with lactose rescued the ESM-mediated decrease in cell viability, indicating lactose-containing glycans in the leukemia cells acted as a counterpart of the ESM for interaction. Taken together, these results suggest that ESM could induce mitochondria-mediated apoptosis through membrane interaction of the K562 human leukemia cells. To the best of our knowledge, this is the first observation that ESM has anti-tumor activity in human cells.

## 1. Introduction

Chronic myeloid leukemia (CML) are well-studied, bone marrow-derived cells. CML was found to be specific for the BCR/ABL fusion oncoprotein, which possesses unusual kinase activity. Because BCR/ABL protein kinase activates many different protein kinases involved in cell survival signaling pathways, thereby inhibiting cell differentiation and increasing the number of undifferentiated cells, the proliferation of CML cells is dysregulated and level of apoptotic cells is decreased, resulting in immortalization [1,2].

Apoptosis is the regulated death of cells, characterized by morphological changes, such as cell shrinking, nuclear condensation, DNA fragmentation and membrane blebbing without any disruption of the plasma membrane [3]. Apoptosis-related signaling is operated by two major pathways, known as intrinsic and extrinsic pathways, and mitochondria also play important roles in both. During apoptosis, the anti-apoptotic Bcl-2 protein family regulates the mitochondrial membrane permeability, maintaining the membrane potential. When apoptosis events are activated by various proaopototic agents, including Fas and tumor necrosis factors-α, the permeabilization of the mitochondrial outer membrane is increased by the induced apoptosis signaling. With immediate apoptosis, cytochrome C is released from the mitochondria to the cytosol, resulting in induction of the apoptosome [4,5,6]. Subsequently, effector caspases such as caspase-3 and caspase-7 are activated after activation of the initiator, caspase-8. Eventually, poly adenosine diphosphate-ribose polymerase (PARP), an inhibitor of DNase, is cleaved by the effector caspases [3,7].

For immunoprotection, the epidermic surfaces of lower animals have constituents of many different mucosaccharides, mucoproteoglycans and carbohydrate-binding lectin proteins [8,9,10]. For example, the skin of eels is covered with carbohydrate-specific proteins, mucus and mucopolysaccharides for innate immune reaction [11,12]. During studies on the proteineous defense mechanism(s), it was recently reported that AJL-2, as a lectin, and the AJN-10 peptide in skin mucus isolated from *A. japonica* have antibacterial activities against *Escherichia coli* K12 and *Aeromonas hydrophila*, respectively [11,13]. In addition, conCL-s lectin in the skin mucus of the conger eel was found to have agglutination activity to *Saccharomyces serevisiae* and *Escherichia coli* K12 [14]. In humans, it was reported that exogenous treatment with galectin-1, which binds to CD7, CD43 and CD45, induced cell death in thymocytes isolated from human surgical specimens, as well as activated human peripheral T cells [15,16]. Similarly, galectin-1 also induced apoptosis in human umbilical vein endothelial cells [17]. In addition, Stillman *et al.*, (2006) reported that the galectin-3 isolated from human cells induced apoptosis in human thymocytes and T cells [18]. However, the functions of isolated eel skin mucus (ESM) in human cells are not well known.

Previously, our group demonstrated that n-hexane extracts from eel bones, internal organs and the entrails of *A. japonica* have anti-inflammatory activity on mast cells and displayed anaphylaxis in *in vitro* cells and an *in vivo* animal model using mice [19]. Additionally, we reported the pharmacological capacity of eel extracts, which resulted in decreased intracellular glucose levels in L6 myotubes and in an animal model [20]. It was suggested that the anti-inflammatory activity of n-hexane extracts might be extended to a biological mechanistic study. Since the ESM was not reported to inhibit or control cancer cells, we extended analysis of the above anti-inflammatory activity to human tumor cells herein, through examination of human leukemia K562 cells.

In the present study, for the first time, we demonstrated that ESM effectively induces apoptosis in human leukemic K562 cells. We also found that treatment with exogenous lactose rescues the ESM-mediated reduced viability of the cells, suggesting that the ESM might be the lactose-binding molecules with antitumor activity.

## 2. Results

### 2.1. Reduction of Cell Viability of Leukemic K562 Cells by ESM

**Figure 1:**
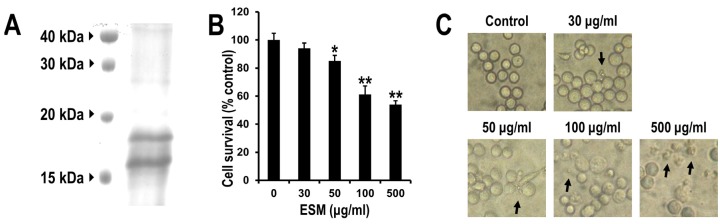
Electrophoretic analysis and effects of ESM on cell viability and morphology of K562 cells. The isolated ESM of 50 μg was resolved by SDS-PAGE (**A**); K562 cells were treated with various concentrations of ESM for 24 h under serum free conditions, after which the cell viability was measured by MTT assay (**B**); and cell morphology was examined by light microscopy (**C**). Magnification 100×. Black arrows indicate the dead cells. The data shown are representative of three independent and indicate mean ± SEM. ** *P* < 0.01 and * *P* < 0.05 compared with control.

In order to investigate the protein properties of ESM, SDS-PAGE analysis was performed. Several minor bands and major bands at 16 and 18 kDa were detected on SDS-PAGE (Figure 1A). Previously, Tasumi *et al.*, (2002) [13] reported the SDS-PAGE patterns of lactose affinity purified fractions of skin mucus isolated from *A. japonica*. In the SDS-PAGE results (Figure 1A), the molecular pattern of ESM was similar to that reported by Tasumi *et al.* (2002) [13].

To date, the pharmacological activity of ESM has not yet been examined on leukemic cells. Thus, to investigate the cytotoxic effects of ESM, the anti-proliferative capacity on human leukemic K562 cells was examined via cell viability assay. When the K562 cells were exposed to ESM in various concentrations, the cell viabilities were decreased in a dose-dependent manner (Figure 1B). Thirty micrograms per milliliter of ESM induced cell death by 5.9% compared to the control (not treated), while 50 μg/mL of ESM exhibited 14.9% growth inhibition. When the cells were treated with 100 μg/mL, the viability was reduced by 39%. Morphological changes were also observed via light microscopy, revealing characteristic changes of the apoptotic cells (Figure 1C). These data suggest that ESM effectively induced cell death in leukemic K562 cells.

### 2.2. Induction of Apoptosis by ESM and Analysis of ESM-Mediated Signaling Pathway

**Figure 2:**
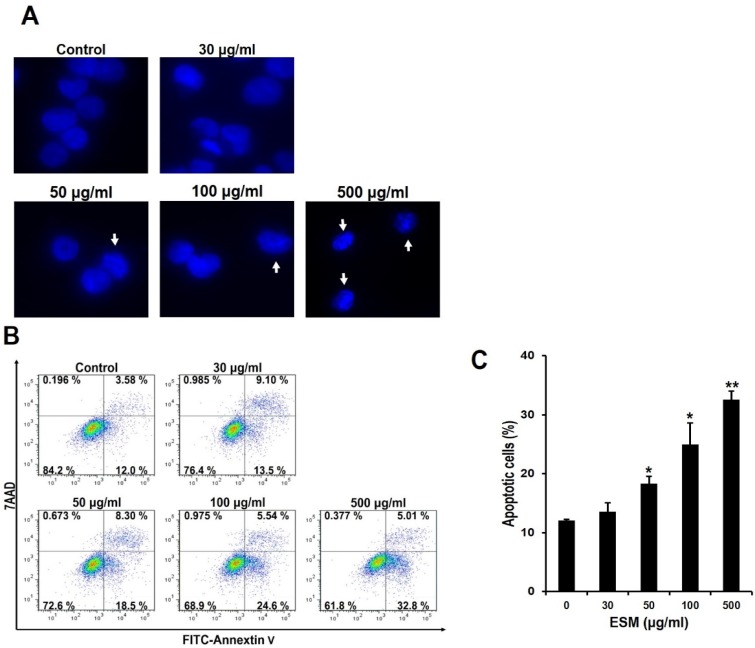
ESM induces apoptosis in K562 cells. After the K562 cells were treated with the indicated concentrations of ESM for 24 h under serum free conditions, cells were stained with DAPI. The morphological features of the nuclei were then determined by fluorescent microscope (**A**). Under the same conditions, after the K562 cells were stained with Annexin V-FITC/7AAD, the Annexin V-FITC and/or 7AAD positive cells were detected by flow cytometry (**B**); Quantitative analysis of the Annexin V-FITC positive cells was then carried out (**C**); Data are presented as the mean ± SEM (*n* = 3). ** *P* < 0.01 and * *P* < 0.05 compared with control.

To determinate which type of cell death was linked to the ESM, the control and ESM-treated cells were stained with DAPI and observed under fluorescence microscope. In contrast to the nuclei of the control group, showing round shapes, the nuclei of the ESM-treated cells displayed high levels of chromatin condensations and nuclear fragmentation (Figure 2A). It is well known that phosphatidylserine and phosphatidylethanolamine, which have high binding affinity for Annexin V, are exposed to the outer membrane during apoptotic cell death [3]. For further confirmation of the effects of ESM on apoptosis of K562 cells, the ESM-treated cells were stained with Annexin V-FITC and the proportion of positively stained cells was detected using flow cytometry. Flow cytometric analysis showed that the number of Annexin V-positive cells increased in a dose-dependent manner (Figure 2B). As summarized in Figure 2B, treatment with 30 and 100 μg/mL of ESM resulted in 13.5% and 24.5% of the cells testing positive for FITC-Annexin V, respectively. Moreover, 500 μg/mL ESM treatment exhibited 32.8% FITC-Annexin V positive cells. These results strongly support the antitumor activity of ESM, as shown in Figure 1. From the results, it is suggested that death of the K562 cells is induced by the ESM.

**Figure 3:**
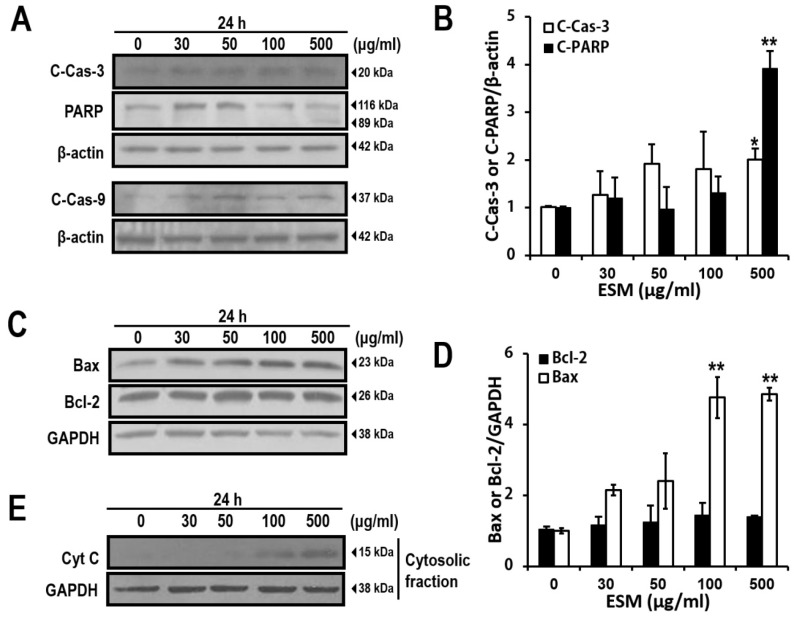
ESM induces apoptosis signaling pathway in K562 cells. ESM-treated cells were analyzed by immunoblotting with antibodies against cleaved PARP (C-PARP, 89 kDa), full length PARP (116 kDa), cleaved caspase-3 (C-Cas-3) and cleaved caspase-9 (C-Cas-9). (**A**) Levels of Bax and Bcl-2 expression were then determined by immunoblotting analysis (**C**); The bar chart shows densitometric analysis of the indicated bands in A and C, respectively (**B**,**D**); Cytochrome C (Cyt C) in the cytosolic fractions was detected by immunoblotting (**E**). β-Actin and GAPDH were used as the internal controls. Data are presented as the mean ± SEM (*n* = 3). ** *P* <0.01 and * *P* < 0.05 compared with control.

To confirm the apoptosis signaling pathways regulated by ESM, investigation of the apoptosis signaling pathways was carried out next. As shown in Figure 3A, when K562 cells were treated with ESM, the cleaved forms of PARP, caspase-3 and caspase-9 were increased. In addition, the Bax expression was also increased in a dose-dependent manner (Figure 3C). However, the expression of Bcl-2, a representative anti-apoptotic signaling protein, was not changed. Furthermore, the amounts of cytochrome c released to the cytosol displayed a dramatic increase after treatment with ESM at the 100 and 500 μg/mL concentrations (Figure 3E). Taken together, these results again support that ESM induces apoptosis in K562 cells.

### 2.3. ERK and p38, But Not JNK Signals, Are Involved in ESM-Mediated Apoptosis

**Figure 4:**
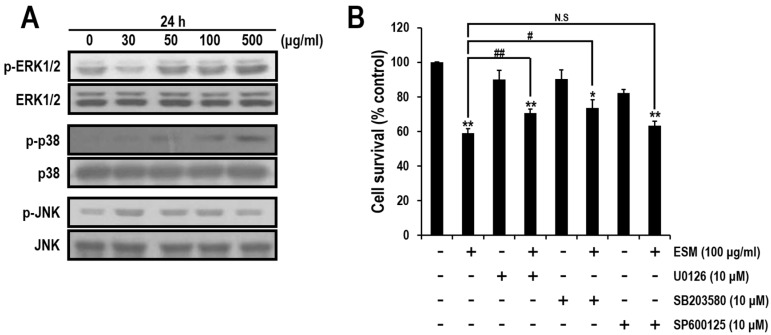
Effects of MAPK inhibitors during ESM-mediated apoptosis. Expression levels of ERK, p-ERK, p38, p-p38, JNK and p-JNK were detected by immunoblotting assays in K562 cells (**A**). The cell viabilities treated with ESM and U0126 or SB203580 or SP600125 were measured by MTT assays (**B**). Three independent data sets are presented as the mean ± SEM. ** *P* < 0.01 and * *P* < 0.05 compared with control. ^##^ *P* < 0.01 and ^#^ *P* < 0.05. N.S indicates not significant.

Mitogen-activated protein kinases (MAPK) signaling plays pivotal roles in apoptosis pathway [21,22]. In order to investigate effects of MAPK during apoptotic cell death by ESM, we performed immunoblotting analysis. Although expression levels of extracellular signal-regulated kinase 1 and 2 (ERK1/2), p38 and c-Jun-NH_2_-terminal kinases (JNK) were not changed, the levels of phospho-ERK1/2 and p38 expression were increased in dose-dependent manners (Figure 4A). To further confirm the signaling pathways regulated by the ERK, p38 and JNK during apoptosis, specific inhibitors of these kinases were used. As shown Figure 4B, viabilities of the co-treated cells with ESM and U0126 (ERK1/2 specific inhibitor) or SB203580 (p38 specific inhibitor) were increased. However, the level of phospho-JNK and viability of the co-treated cells with ESM and SP600125 (JNK specific inhibitor) cells were not significantly changed (Figure 4B). These results suggested that ERK1/2 and p38 signals are involved in the ESM mediated-apoptosis.

### 2.4. Lactose Suppresses Reduction of Cell Viability by ESM

It is well known that ESM includes a variety of lectins [9,23]. As shown in Figure 1A, the protein properties were characterized. Thus, it was regarded that lectin-like molecules in ESM are the potential apoptosis-inducing factors acting on K562 cells. Therefore, we further characterized the ESM by MTT assays wherein K562 cells were treated with several mono- and disaccharides that act as antagonists between the lectins in ESM and the carbohydrate residues on the surfaces of the K562 cells. Although no significant change in the viabilities of cells co-treated with ESM and either galactose or glucose were observed compared with the control group of ESM treatment alone, the viabilities of lactose co-treated cells were higher than the control group (Figure 4). These results indicated that the biologically active protein in the ESM might be a lectin like protein. Since the AJL-1 and -2 C-type lectins are known to strongly bind to lactose [6], it is suggested that lactose binding lectin-like molecules in ESM may be the apoptosis-inducing factors.

## 3. Discussion

To protect themselves, fish have many humoral factors, such as antimicrobial peptides and proteins, proteases, lysozyme, complement and lectins [18]. Especially, the lectins secreted by the club cells of the eel epithelium play important roles in the innate immunity through recognition and opsonization of pathogens [3,9,14]. In the fields of aquaculture and fish immunology, a lot of research has been performed on the humoral factors of fish.

Previously, our group demonstrated that n-hexane extracts from eels specifically inhibit prostaglandin D2 generation through selective inhibition of the cyclooxygenase-2 and arachidonic acid metabolic pathways in mouse bone marrow-derived mast cells and a mouse model [19]. Ethylacetate extracts of *A. japonica* were also found to increase the AMPK activity for sensing the cellular energy status and glucose uptake in L6 myotubes [20]. The eel extract-mediated molecular signaling improved the glucose and lipid profiles in *db*/*db* mice, displaying anti-diabetic activities. These improved glucose and lipid profiles in diabetic *db*/*db* mice resulted from AMPK activity in the muscle tissues [23].

Lectins are carbohydrate recognition proteins that play many roles in biological functions through the recognition of specific glycan moieties [9,24,25]. To date, an increasing number of lectins have been reported from plant, insect and animal sources [23,24,25]. There are several known kinds of lectins, including C-type, sialic acid-binding immunoglobulin-like lectins, galectins and heparin-binding proteins [9,26]. Fish lectins are also presented in ESM, for instance, congerin I and II, as galectin-recognizing β-galactoside-containing glycans, isolated from conger eel, *Conger myriaster* [27,28]. AJL-1 and -2 purified from *A. japonica* [13], the C-type lectins that bind to lactose, are also well known. Interestingly, the two lactose-specific lectins of AJL-1 and AJL-2 were isolated from the skin mucus of the Japanese eel, *A. japonica*, [29,30] and they were characterized as the galectin family. More specifically, AJL-1 was classified as a defensive factor, while AJL-2 agglutinated and suppressed cell growth of *E. coli* K12. Our lactose-binding lectin, ESM, was isolated from eel species that are considered to be similar to the Japanese eel, *A. japonica*. Therefore, the relationship between ESM and AJL types regarding the molecular structure and carbohydrate specificity will be highly interesting in the host defense and anti-tumor activity.

ERK plays roles in cell survival by activating anti-apoptotic functional proteins such as CREB and NF-κB transcription factors [31,32]. However, it was previously reported that during anti-cancer drugs mediated-apoptosis, activation of ERK could trigger cell death by reducing mitochondrial membrane potential and mitochondrial respiration, which could promote cytochrome C release [33,34]. The activation of ERK could increase the expression of Bax, which is a proapoptotic protein [35]. Role of p38 MAPK is also well known in apoptosis signaling pathway. For instance, activation of p38 is induced by treating with anti-cancer drugs in breast cancer cells [36].

**Figure 5:**
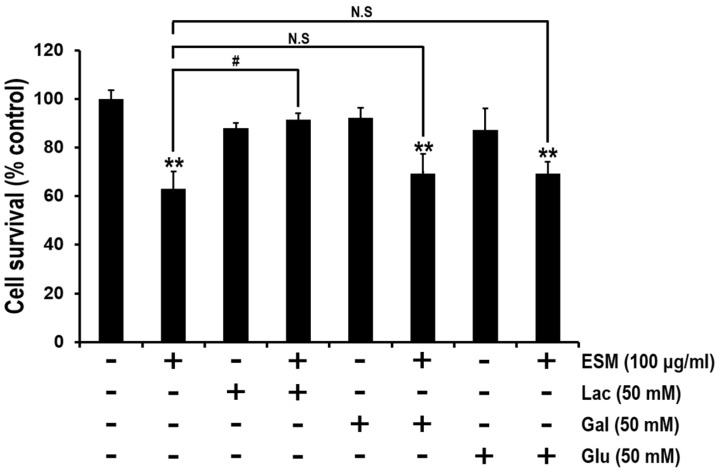
Lactose, but not galactose or glucose, rescues the ESM-mediated decrease of cell viability. In serum free medium, K562 cells were pre-treated with lactose, galactose or glucose for 30 min, respectively, and then treated with ESM for 24 h. Subsequently, cell viabilities were measured by MTT assay. N.S indicates not significant. Three independent data sets indicate mean ± SEM. ** *P* < 0.01 compared with control; ^#^ *P* < 0.05 compared with ESM-treated group.

**Figure 6:**
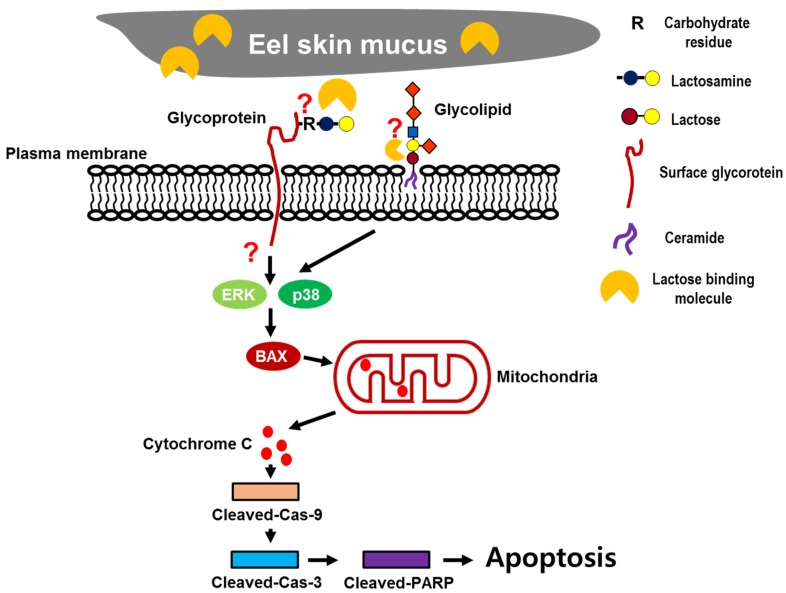
Schematic diagram of the mechanisms of ESM-mediated apoptosis.

In the present study, to extend the pharmacological capacities of eel extracts, we successfully isolated and partially purified the ESM. For the first time, we demonstrated that the ESM triggers apoptotic cell death and inhibits cell proliferation in human leukemic K562 cells (Figure 1 and Figure 2). Our results have clearly shown that the cleaved forms of caspase-9, caspase-3 and PARP were also found to be increased in dose-dependent manners (Figure 3A,B). In addition, the expression of Bax and release of cytochrome C into the cytoplasm, as apoptotic signals, were dose-dependently increased by treatment of K562 cells with ESM (Figure 3C–E). Furthermore, phosphorylation levels of ERK1/2 and p38 in ESM-treated K562 cells were increased (Figure 4A). When cells were co-treated with ESM and inhibitor of ERK1/2 or p38, the cell viabilities were increased (Figure 4B). These results suggest that ESM induce apoptosis via activations of ERK1/2 and p38. Interestingly, when the K562 cells were co-treated with lactose, the reduction of cell viability due to ESM treatment was recovered (Figure 5). These findings suggest that lactose-binding molecules in ESM are likely the factors inducing apoptosis in K562 cells, as summarized in Figure 6. It is considered that ESM has similar protein properties compared with the lectins of other groups (Figure 1A) [13,14]. Thus, these molecules should be further studied. Our findings may provide a new drug candidate as an alternative therapy for human leukemia in the future.

## 4. Materials and Methods

### 4.1. Preparation of Eel Skin Mucus

Korean eels were purchased from a commercial supplier. The skin mucus was gathered by scraping the surface of ten healthy eels, weighing from 250 to 400 g, after which the mucus was homogenized with 2 volumes of 0.01 M phosphate buffered saline (PBS). After the mucus was centrifuged at 13,000 rpm for 30 min at 4 °C, the supernatant was lyophilized. Dried substance was weighed and dissolved in PBS to the final concentration of 10 mg/mL. Then, the dissolved substance was filtered using a 50-mL syringe and a 0.2-μm syringe filter (Sartorius Stedim Biotech, Göttingen, Germany) and it was used as the ESM.

### 4.2. Cell Culture and Reagents

The human chronic leukemia cell line K562 was cultured in RPMI 1640 (WelGENE Co., Daegu, Korea) supplemented with 10% fetal bovine serum, 100 U/mL penicillin and 100 U/mL streptomycin. The cells were maintained in 5% CO_2_ in a humidified incubator at 37 °C. d(+)-galactose and lactose were purchased from Junsei Chemical Co. (Tokyo, Japan). d(+)-glucose was obtained from Sigma-Aldrich (St. Louis, MO, USA). U0126 (ERK1/2 inhibitor), SB203580 (p38 inhibitor) and SP600125 (JNK inhibitor) were purchased from Merck Millipore (Billerica, MA, USA).

### 4.3. MTT Assay and Light Microscopic Analysis

K562 cells (1 × 10^4^ cell/100 μL) were seeded in 96-well plates in serum free conditions and treated with 0–500 μg/mL ESM for 24 h. The cells were incubated with 3-(4,5-dimethylthiazol-2-yl)-2,5-diphenyltetrazolium bromide (MTT) solution for 3 h. After incubation for 3 h, the supernatant was removed, the formazan crystals were dissolved in DMSO, and measurement was carried out at 490 nm with a microplate reader. Under the same conditions, morphological changes of the K562 cells were visualized using light microscopy (Nikon Eclipse 80i).

### 4.4. Immunoblot and Densitometric Analysis

Polyclonal rabbit anti-human Bax, polyclonal rabbit anti-human Bcl-2, polyclonal rabbit anti-human JNK, monoclonal mouse anti-human p38, monoclonal mouse anti-human p-JNK and polyclonal goat anti-human cytochrome C antibodies were purchased from Santa Cruz Biotechnology (Santa Cruz, CA, USA). Monoclonal mouse anti-human glyceraldehyde-3-phosphate dehydrogenase (GAPDH) antibody was obtained from Millipore (Milford, MA, USA). Polyclonal rabbit anti-human β-actin antibody was purchased from Sigma-Aldrich. Polyclonal rabbit anti-human PARP, anti-human cleaved caspase-3, anti-human cleaved caspase-9, anti-human p-ERK and anti-human p-p38 as well as monoclonal mouse anti-human ERK antibodies were obtained from Cell Signaling Technology (Dancers, MA, USA). The secondary antibodies were horseradish peroxidase conjugated anti-mouse IgG, anti-rabbit IgG (Thermo Scientific Pierce, Rockford, IL, USA) and anti-goat IgG (Santa Cruz Biotechnology, CA, USA).

Cells were rinsed twice with PBS and lysed in RIPA lysis buffer containing 20 mM Tris/HCl pH 7.5, 150 mM NaCl, 1% Triton X-100, 2 mM EDTA, 10% glycerol, 0.1% SDS and 0.5% sodium deoxycholate with protease inhibitor cocktail, including 1 mM Na3VO4, 20 μg/mL PMSF, 10 μg/μL leupeptin and 50 mM NaF. Lysates were centrifuged at 13,000 rpm for 10 min and pellets were removed. For cytosolic fractions, cytosol was purified using a mitochondrial fractionation kit, following the recommended protocol (Activemotif, Carlsbad, CA, USA). Concentrations of the whole cell lysate and cytosolic fraction were measured using Lowry assay (Bio-Rad, Hercules, CA, USA). Thirty micrograms of whole cell lysates and cytosolic fraction were separated by sodium dodecyl sulfate-polyacrylamide gel electrophoresis (SDS-PAGE) and transferred onto PVDF membranes using the Hoefer electrotransfer system (Amersham Biosciences, Amersham, UK). The transferred PVDF membranes were incubated with the above-mentioned primary antibodies (1:1000 dilution in 1% skim milk) at 4 °C overnight. And the membranes were incubated with secondary antibody (1:5000 dilution in 1% skim milk) for 2 h at room temperature. Bands were visualized by enhanced chemiluminescence (Sigma-Aldrich) and exposed to X-ray film. The X-ray films were scanned onto a computer, and densitometric analysis of bands was performed by Image J software.

### 4.5. 4′,6-Diamidino-2-phenylindole (DAPI) Staining

After treatment with ESM, cells were washed twice with PBS, fixed with 4% paraform-aldehyde in PBS for 20 min and permeabilized in 0.1% triton X-100. The cells were then stained with DAPI for 20 min at room temperature. After staining, cells were added to mounting medium (Dako, Glostrup, Denmark) and observed under a fluorescence microscope (Nikon, Tokyo, Japan).

### 4.6. Annexin V-FITC/7-Amino-Actinomycin (7AAD) Staining and Analysis by Flow Cytometry

For detection of apoptotic cells, K562 cells were treated with ESM at the indicated concentrations for 24 h. After treatment, the cells were washed twice with PBS and stained with 1 mg/mL of Annexin V- FITC and 7AAD (BD Pharmingen) at room temperature in the dark and analyzed on a flow cytometer (FACS Canto II).

### 4.7. Statistical Analysis

The results of the MTT assay and densitometric analysis were analyzed by Student’s *t*-test. *P*-value of less than 0.05 was considered statistically significant.
